# Comparison of MLC error sensitivity of various commercial devices for VMAT pre‐treatment quality assurance

**DOI:** 10.1002/acm2.12288

**Published:** 2018-03-03

**Authors:** Masahide Saito, Naoki Sano, Yuki Shibata, Kengo Kuriyama, Takafumi Komiyama, Kan Marino, Shinichi Aoki, Kazunari Ashizawa, Kazuya Yoshizawa, Hiroshi Onishi

**Affiliations:** ^1^ Department of Radiology University of Yamanashi Yamanashi Japan

**Keywords:** MLC error, patient QA, radiotherapy, VMAT

## Abstract

The purpose of this study was to compare the MLC error sensitivity of various measurement devices for VMAT pre‐treatment quality assurance (QA). This study used four QA devices (Scandidos Delta4, PTW 2D‐array, iRT systems IQM, and PTW Farmer chamber). Nine retrospective VMAT plans were used and nine MLC error plans were generated for all nine original VMAT plans. The IQM and Farmer chamber were evaluated using the cumulative signal difference between the baseline and error‐induced measurements. In addition, to investigate the sensitivity of the Delta4 device and the 2D‐array, global gamma analysis (1%/1, 2%/2, and 3%/3 mm), dose difference (1%, 2%, and 3%) were used between the baseline and error‐induced measurements. Some deviations of the MLC error sensitivity for the evaluation metrics and MLC error ranges were observed. For the two ionization devices, the sensitivity of the IQM was significantly better than that of the Farmer chamber (*P* < 0.01) while both devices had good linearly correlation between the cumulative signal difference and the magnitude of MLC errors. The pass rates decreased as the magnitude of the MLC error increased for both Delta4 and 2D‐array. However, the small MLC error for small aperture sizes, such as for lung SBRT, could not be detected using the loosest gamma criteria (3%/3 mm). Our results indicate that DD could be more useful than gamma analysis for daily MLC QA, and that a large‐area ionization chamber has a greater advantage for detecting systematic MLC error because of the large sensitive volume, while the other devices could not detect this error for some cases with a small range of MLC error.

## 1 INTRODUCTION

Volumetric arc therapy (VMAT) is commonly used to produce complicated dose distributions using a variable dose rate, multi‐leaf collimator (MLC) shape, and variable gantry speed. Although this technique can help reduce the dose administered to organs at risk, it requires an understanding of each parameter's error on the final dosimetric effect. In particular, the accuracy of MLC movement greatly affects the delivered dose. Oliver et al.,^1^ reported the effects of various MLC error patterns on clinical VMAT plans, and found that MLC error was linearly correlated with the generalized equivalent uniform dose for all error types. Therefore, quality assurance (QA) is needed to address MLC positioning errors.

Several studies have attempted to examine this issue. One approach to MLC QA is to analyze the machine's logfile to perform MLC QA,^2^, ^3^ and this approach can be used with data from a dynamic delivery (MLC position, Gantry Angle, and Monitor Unit) with higher sampling rate (10–50 ms). In addition, logfile QA can be performed using three‐dimensional dose reconstruction based on patient geometry, and several recent reports have described this technique.^4^, ^5^, ^6^

The second MLC QA method is based on measurements that are obtained using various radiation detectors, which can involve an electric portal imaging device (EPID),^7^ a film or a two‐dimensional (2D) diode (or chamber) array,^8^ or a 3D diode array.^9^ A gamma analysis was developed by Low et al.,^10^ which combined the dose difference (DD) and distance to agreement (DTA). Although 2D gamma analysis has been used in clinical situations, 3D gamma analysis has been used for volume dose analysis, such as comparing phantom and patient doses.^11^ Although the gamma pass rate is usually used in clinical situations, it is not an absolute evaluation index, and Hussein et al. demonstrated that different device and software combinations exhibited variable agreement with the predicted values for the same pass rate criteria.^12^ Furthermore, Nelms et al. reported that gamma pass rates were not strongly correlated with dose errors in anatomical regions of interest.^13^ Therefore, to predict the real patient dose, the measurement dose reconstruction method has been developed.^14^

Although there is increasing awareness of the importance of irradiation QA, the importance of accurately monitoring MLC movement/position remains unchanged, as it affects the accuracy of dose prescription. Some studies have investigated the MLC error sensitivity of different QA devices.^8^, ^15^ However, there remains insufficient evidence regarding the advantages or disadvantages of each QA device in the clinical setting. Furthermore, a large area ionization chamber has not been investigated. Therefore, this study aimed to compare the MLC error sensitivity of various measurement devices for VMAT pre‐treatment QA.

## 2 METHODS

### 2.A QA devices

**Figure 1:**
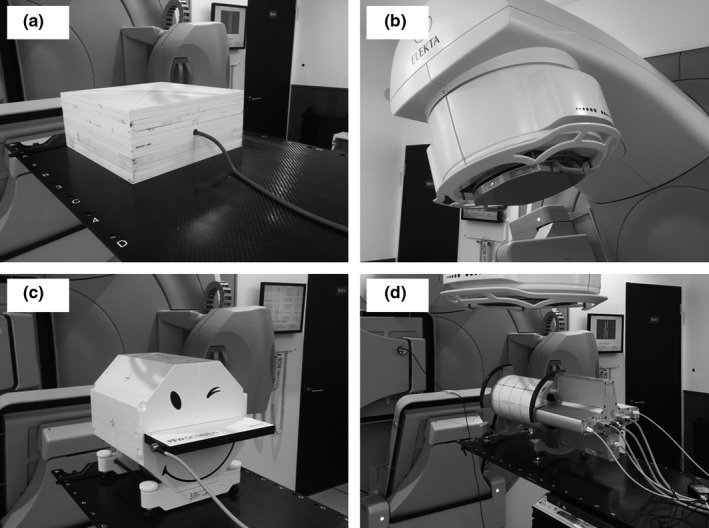
Phantom setup for each device ((a)–(d)). A Farmer chamber was used by inserting to the solid phantom (a). The IQM was used by mounting on the linac gantry head (b). The 2D‐array (c) was used by inserting to an octagon‐shaped phantom (Octavius Phantom) with a central cavity.

This study used four QA devices (Delta4: ScandiDos, Uppsala, Sweden; 2D‐array seven29: PTW, Freiburg, Germany; IQM: iRT Systems GmbH, Koblenz, Germany; Farmer chamber: PTW 30013). Figure 1 shows the phantom setup for each device. The Delta4 device is a cylindrical PMMA phantom that surrounds two crossing orthogonal planes with 1,069 p‐Si diodes. The diodes are disc‐shaped, have a volume of 0.04 mm^3^, and are placed at 5‐mm intervals in the central areas (6 cm × 6 cm) and at 10‐mm intervals in the outer areas (up to 20 cm × 20 cm). The 2D‐array is equipped with 729 equally spaced ionization chambers, with a center‐to‐center distance of 1 cm and covering an active area of 27 cm × 27 cm. Each chamber has a size of 5.0 × 5.0 × 5.0 mm. An octagon‐shaped phantom (Octavius Phantom) with a central cavity was used to insert the 2D ion chamber array.

An integral quality monitoring system (IQM) is a large‐area ionization chamber that was introduced by Islam et al.^16^ The IQM is usually mounted on the linac gantry head, has aluminum components, and provides a sensitive area of 22 cm × 22 cm. The chamber can monitor a radiation field that projects to a size of approximately 34 cm × 34 cm at the isocenter. The sensitive volume of the ion chamber was approximately 530 cm^3^. To produce greater spatial sensitivity, the optimized inclined detection plane was used, and additionally technical details have been described in a previous report.^16^ The 0.6‐cc Farmer chamber was used by inserting a solid water phantom (30 × 30 × 20 cm) to a depth of 10 cm.

### 2.B MLC error plans

Nine retrospective VMAT plans were used: three head and neck boost plans (30 Gy/15 fractions), three lung stereotactic body radiation therapy (SBRT) plans (50 Gy/4 fractions), and three prostate plans (76 Gy/38 fractions). All plans were generated as half arcs, full arcs, or two full arcs using the Pinnacle3 treatment planning system (Philips Radiation Oncology Systems, Madison, WI, USA) and the SmartArc optimization algorithm. The calculations were performed with a dose calculation grid of 2 mm and control points of 2°. The irradiation was performed using a 6‐MV photon beam that was generated by a Synergy linier accelerator (Elekta Oncology Systems, UK) and an Agility gantry head, which has 160 multi‐leaf collimators (width: 5 mm).

**Figure 2:**
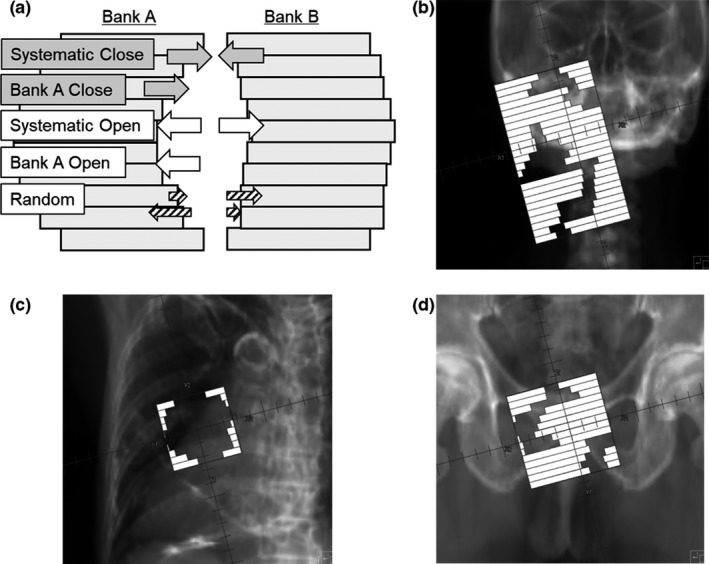
The MLC error patterns (a) and typical apertures of the original plan for each treatment site (head and neck (b), lung‐SBRT (c), and prostate (d)).

To investigate the MLC error sensitivities, nine MLC error plans were generated for all nine original VMAT plans. First, we investigated the systematic open and close errors. The errors were introduced on each side of the bank using an in‐house C++ program, and the error patterns were determined as systematic open (0.75, 0.50, and 0.25 mm) and systematic closed (–0.25, –0.50, and –0.75 mm) for each bank. In addition, bank A open and close error (1 mm for each) and random error (*σ *= 0.5 mm) were also investigated. The MLC error patterns and typical apertures of the original plan for each treatment site are shown in Fig. 2.

### 2.C Evaluation of MLC error sensitivity

The MLC error sensitivity for each device was evaluated by comparing the doses of the original VMAT plan to that of the corresponding error plan. To investigate the sensitivity of the Delta4 device and the 2D‐array, global gamma analysis (1%/1, 2%/2, and 3%/3 mm), DD (1%, 2%, and 3%) were used. The analyses were performed using 20% of the maximum dose as the low dose threshold, and pass rates (%) were calculated for all analyses. The IQM and Farmer chamber were evaluated using the cumulative signal difference (*S*_*diff*_) which was calculated using the following equation:

(1) $$S_{\mathrm{diff}}\left[\%\right]=\left(\frac{S_{\mathrm{error}}-S_{\mathrm{baseline}}}{S_{\mathrm{baseline}}}\right)\times 100$$

In this equation, *S*_*baseline*_ and *S*_*error*_ are the cumulative signals of the baseline plan and the MLC error plan, respectively. The Wilcoxon signed‐rank test was used to compare IQM versus the Farmer chamber and the 2D‐array versus the Delta4 device. All analyses were performed using JMP Pro software (version 11; SAS Institute Inc., NC).

## 3 RESULTS

**Figure 3:**
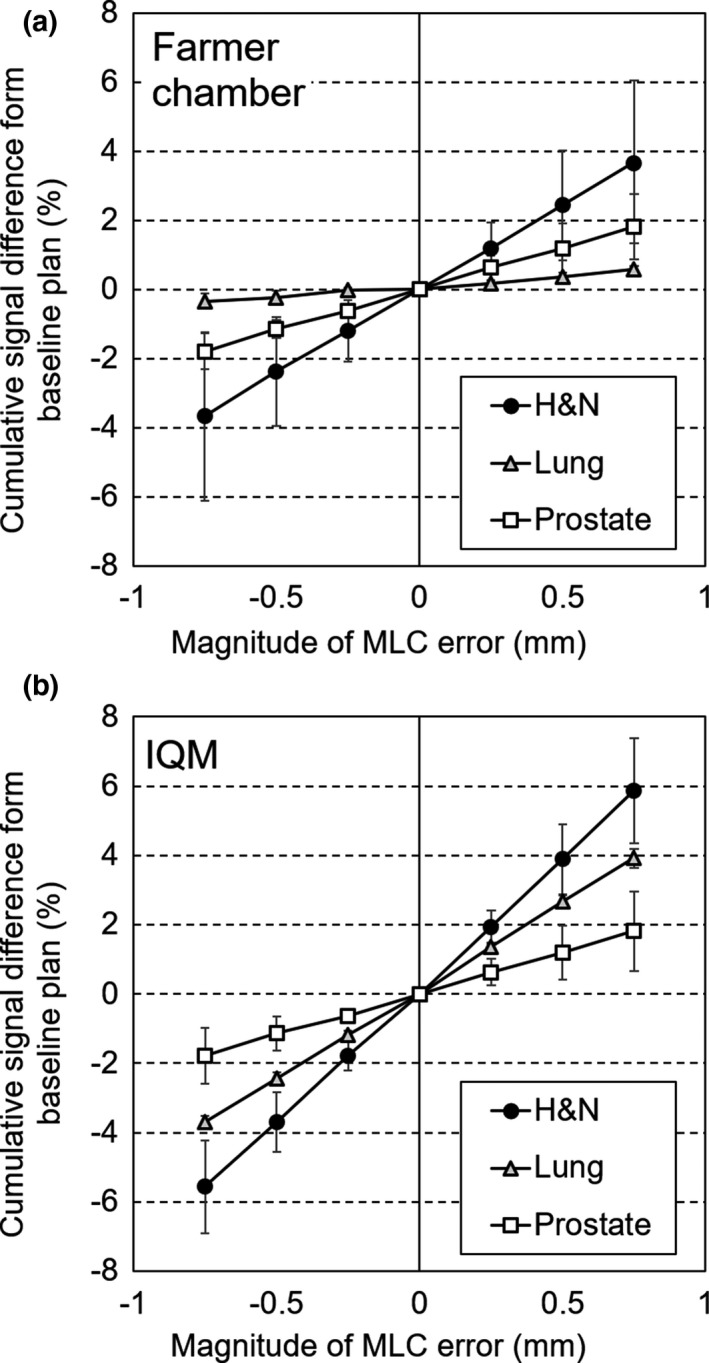
Cumulative signal differences between the baseline and systematic open/closed MLC error plans for each treatment site. The results of IQM (a) and Farmer chamber (b) are represented. The results for the IQM and Farmer chamber had a good linear correlation (*R* ^*2*^>0.99) between the cumulative signal difference and the magnitude of the MLC errors.

Figure 3 shows the cumulative signal differences between the baseline and systematic open/closed MLC error plans for each treatment site. The results for the IQM and Farmer chamber had a good linear correlation between the cumulative signal difference and the magnitude of the MLC errors. The signal differences between the baseline and MLC error plans for the Farmer chamber and IQM are summarized in Table 1. The average values and standard deviations for all nine plans are listed, and the sensitivity of the IQM was significantly better than that of the Farmer chamber (*P* < 0.01) when excluding the random error.

**Table 1:** Signal differences (*S*_*diff*_) between the baseline and nine MLC error plans for the Farmer chamber and IQM. The average values and standard deviations for all nine plans are represented. The *P*‐values are calculated by the Wilcoxon test

Error type	Magnitude of MLC error (mm)	Diff (%, mean ± SD)		
		Farmer chamber	IQM	*P* ‐value
Random	*σ *= 0.50	0.31 ± 0.99	0.14 ± 0.18	0.250
Bank A open	1.00	1.58 ± 1.03	3.16 ± 0.86	0.008
Bank A close	1.00	−0.98 ± 1.86	−2.81 ± 0.90	0.020
Systematic open/close	−0.75	−1.78 ± 2.05	−4.67 ± 1.19	0.008
	−0.50	−1.14 ± 1.32	−3.09 ± 0.77	0.008
	−0.25	−0.55 ± 0.72	−1.50 ± 0.37	0.008
	0.25	0.63 ± 0.66	1.65 ± 0.43	0.004
	0.50	1.30 ± 1.33	3.30 ± 0.90	0.004
	0.75	1.96 ± 1.98	4.94 ± 1.37	0.004

**Figure 4:**
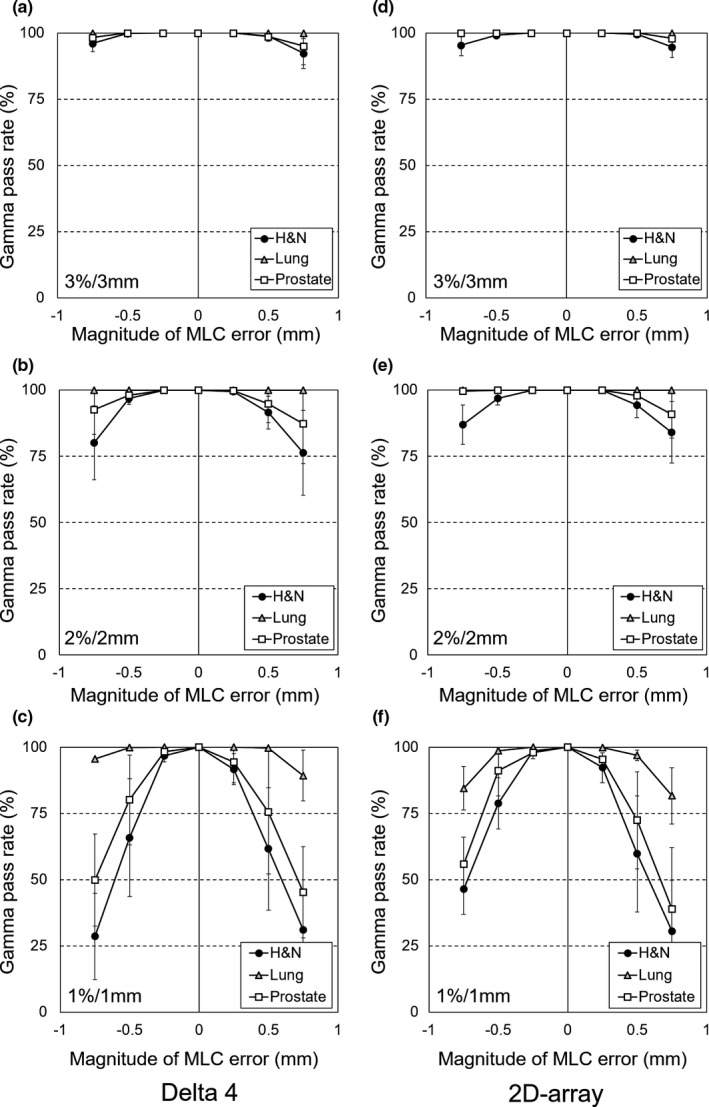
The gamma pass rates between the baseline and MLC error plans for the Delta4 device and the 2D‐array using the three criteria (1%/1, 2%/2, and 3%/3 mm). The results of Delta4 ((a)–(c)) and 2D‐array ((d)–(f)) are represented.

Figure 4 shows the gamma pass rates between the baseline and systematic open/closed MLC error plans for the Delta4 device and the 2D‐array using the three criteria (1%/1, 2%/2, and 3%/3 mm). The pass rates decreased as the magnitude of the MLC error increased for both devices. In particular, the gamma analysis using the strictest criteria (1%/1 mm) had better sensitivity than the loosest criteria (3%/3 mm). Furthermore, the small MLC error for small aperture sizes, such as for lung SBRT, could not be detected using the loosest gamma criteria (3%/3 mm). Tables 2 and 3 shows the gamma pass rates and DD between the baseline and error plans for the 2D‐array and Delta4 device. The DD provided better sensitivity than the gamma analyses, although there were no significant differences between the two devices for almost all cases.

**Table 2:** Gamma pass rate and dose difference between a baseline and the nine error plans for a 2D‐array and Delta4 for all nine plans. Three criteria of gamma pass rate (1%/1, 2%/2, and 3%/3 mm) are represented. The *P*‐values are calculated by the Wilcoxon test

Error type	Magnitude of MLC error (mm)	Pass rate (%, mean ± SD)								
		1%/1 mm			2%/2 mm			3%/3 mm		
		Delta4	2D‐array	*P*‐value	Delta4	2D‐array	*P*‐value	Delta4	2D‐array	*P*‐value
Random	σ = 0.50	95.8 ± 9.1	97.3 ± 3.3	1.000	98.5 ± 4.2	99.8 ± 0.6	1.000	99.5 ± 1.5	100.0 ± 0.1	1.000
Bank A open	1.00	68.1 ± 17.7	79.0 ± 16.6	0.203	91.4 ± 11.0	96.6 ± 3.9	0.297	97.5 ± 5.6	99.1 ± 1.4	0.438
Bank A close	1.00	81.8 ± 16.4	80.0 ± 15.0	0.820	98.7 ± 1.7	97.0 ± 4.2	0.578	99.8 ± 0.3	99.1 ± 1.6	0.438
Systematic open/close	−0.75	54.3 ± 33.1	64.9 ± 27.3	0.652	89.0 ± 15.4	96.8 ± 6.2	0.156	97.7 ± 3.4	99.6 ± 1.0	0.250
	−0.50	79.1 ± 24.6	89.6 ± 15.3	0.301	97.9 ± 3.0	99.6 ± 1.0	0.250	99.9 ± 0.1	100 ± 0.0	0.500
	−0.25	98.0 ± 2.8	99.7 ± 0.8	0.250	100 ± 0.0	100 ± 0.0	1.000	100 ± 0.0	100 ± 0.0	1.000
	0.25	99.4 ± 8.0	89.5 ± 12.5	0.438	99.7 ± 0.5	99.0 ± 1.4	0.313	100 ± 0.1	100 ± 0.0	1.000
	0.50	75.4 ± 28.8	62.5 ± 27.5	0.301	94.6 ± 7.7	91.8 ± 10.9	0.563	99.0 ± 1.6	98.3 ± 2.7	0.438
	0.75	48.8 ± 30.0	28.3 ± 20.7	0.203	85.2 ± 19.4	85.3 ± 16.3	0.844	94.9 ± 7.3	92.2 ± 10.8	0.469

**Table 3:** Dose difference between a baseline and the nine error plans for a 2D‐array and Delta4 for all nine plans. Three criteria of dose difference (1%, 2%, and 3%) are represented. The *P*‐values are calculated by the Wilcoxon test

Error type	Magnitude of MLC error (mm)	Dose difference (%, mean ± SD)								
		1%			2%			3%		
		Delta4	2D‐array	*P*‐value	Delta4	2D‐array	*P*‐value	Delta4	2D‐array	*P*‐value
Random	*σ *= 0.50	93.5 ± 11.0	98.3 ± 1.9	0.219	98.2 ± 4.9	99.9 ± 0.2	0.625	99.4 ± 1.7	100.0 ± 0.1	1.000
Bank A open	1.00	44.9 ± 20.4	85.8 ± 7.6	0.004	76.2 ± 14.0	94.5 ± 5.1	0.004	90.4 ± 8.4	97.8 ± 2.8	0.020
Bank A close	1.00	51.8 ± 16.4	87.7 ± 7.7	0.004	83.6 ± 8.7	96.1 ± 3.9	0.008	93.2 ± 4.9	98.6 ± 1.9	0.020
Systematic open/close	−0.75	19.6 ± 20.1	30.5 ± 23.7	0.734	55.2 ± 26.5	73.5 ± 20.7	0.074	78.2 ± 19.0	90.2 ± 16.0	0.203
	−0.50	39.7 ± 26.5	53.1 ± 30.3	0.496	78.4 ± 17.9	91.9 ± 12.2	0.098	92.2 ± 9.1	98.5 ± 3.0	0.129
	−0.25	78.4 ± 16.7	97.3 ± 4.1	0.055	96.6 ± 4.5	100 ± 0.0	0.031	99.7 ± 0.9	100 ± 0.0	1.000
	0.25	78.6 ± 18.1	73.1 ± 23.5	0.820	97.9 ± 3.1	97.2 ± 4.0	0.887	99.5 ± 1.0	99.9 ± 0.3	0.500
	0.50	42.7 ± 24.9	35.2 ± 21.4	0.496	78.3 ± 19.0	77.2 ± 22.2	1.000	92.8 ± 9.0	92.7 ± 11.1	1.000
	0.75	19.7 ± 17.8	12.5 ± 12.6	0.359	57.2 ± 25.7	57.6 ± 23.7	1.000	79.2 ± 18.2	79.7 ± 21.2	1.000

## 4 DISCUSSION

The accuracy of MLC position and movement affects dose prescription accuracy, and MLC QA is needed for complicated treatments, such as intensity‐modulated radiation therapy. Therefore, AAPM TG‐142 report mentioned that the leaf position repeatability should be within ±1 mm.^17^ Some reports have evaluated the sensitivities of various QA devices to MLC error. For example, Vieillevigne et al. investigated the sensitivities of three devices (ArcCHECK, 2D‐array, and EPID) to gantry error and MLC error, and they concluded that pretreatment QA may not identify delivery errors, even using strict criteria (2%/2 mm).^18^ In addition, Oliver et al. reported that open/closed MLC errors tended to have a greater effect on the clinical dose, compared to random or systematic MLC shift errors.^1^ Our results are consistent with those findings, and indicate that the sensitivity to MLC open/closed error could be better than that to random error for all devices. Thus, the MLC error sensitivity could vary according to the magnitude of the MLC error as well as the treatment site. This study examined three treatment sites (head and neck, lung SBRT, and prostate), with the most complicated delivery needed for the head and neck, which requires a small segmented aperture size and high leaf speed. The treatment field for lung SBRT was relatively simple and used a broad aperture [Fig. 2(c)], as the target shape is spherical and the SmartArc leaf speed is set relatively slow at our institution (1.0 cm/degree). Thus, as a test for the error sensitivity of each device, we used the lung SBRT plan because it would not be significantly affected by MLC open error.

This study compared four different QA devices that had different dosimetry characteristics. For example, the resolutions were different, as the IQM and Farmer chamber had ionization spaces according to each detector's size, while the other devices had a course resolution because the Delta4 and 2D arrays used a diode detector. Kadoya et al. evaluated the MLC error sensitivity using ArcCHECK, which had a resolution of 0.8 × 0.8 mm^2^.^19^ Despite having the same magnitude of MLC error as this study, the dosimetric error was correlated with the MLC error magnitude. Therefore, we used a magnitude of MLC error ranging from –0.75 to 0.75 mm. The ionization devices (the IQM and Farmer chamber) exhibited a linear correlation between the magnitude of the open/closed MLC error and the signal difference. Furthermore, excluding random MLC error, the MLC error sensitivity of IQM was significantly better than that of the Farmer chamber (Table 1), as the sensitive ionization volume was larger and the optimized inclined detection plane was used.^16^ Although IQM is generally used for intra‐treatment validation as a transmission type detector, our study demonstrated that this device could be also useful for daily MLC QA.

Gamma analysis is the most commonly used method in clinical pre‐treatment patient QA for VMAT. Pullium et al. have compared 2D‐gamma and 3D‐gamma analyses, and concluded that 3D‐gamma analysis provided up to 2.9% more pixels passing than 2D‐gamma analysis.^11^ Therefore, we used 2D‐gamma analysis because it is more sensitive than 3D‐gamma analysis. Based on the results from Fig. 4 and Table 2, there were no significant differences between two devices (Delta4 and 2D‐array) for all criteria. It is possible that their course detector resolution affected the pass rates, which could enhance the sensitivities of these devices to MLC error, especially using the loosest criteria (ie, 3%/3 mm). In addition, for the lung SBRT plans, the conventional gamma criteria (2%/2 mm or 1%/1 mm) could not detect small MLC errors, based on a magnitude of ±0.25 mm (Fig. 4). Thus, our results suggest that gamma analysis using loose criteria might be not suitable for detecting relatively small MLC errors when 2D gamma analysis was used, although it may be more sensitive to large MLC errors. However, DD had a better sensitivity than gamma analysis. In addition, the DD of Delta4 had significantly better sensitivity than that of the 2D‐array for some error patterns, as shown in Table 3. Although further studies are needed to evaluate the effects of the detector number (Delta4: 1,069; 2D‐array: 729), our results highlight the usefulness of DD for daily MLC QA.

The 3D patient dose reconstruction system has recently been developed to clarify the effect of dosimetric error on patient geometry. For example, Olch et al. evaluated the accuracy of a 3D dose reconstruction system versus chamber and film using 15 intensity‐modulated radiation therapy cases.^20^ In addition, Saito et al. compared the 3D dose reconstruction system software and logfile based 3D dose reconstruction for various treatment sites.^6^ However, this study only evaluated each QA device's sensitivity to MLC error, although we hope to evaluate the effects of these sensitivities on patient dose using a 3D‐reconstruction system.

The other limitations of this study are its small sample size for each treatment site and the use of only MLC error. Future studies should evaluate more cases and machine‐related errors, such as gantry angle, collimator angle, and dose rate. In addition, we could not investigate the sensitivities of EPID and film, although some previous reports have described MLC QA or patient QA using EPID dosimetry.^21^, ^22^, ^23^, ^24^, ^25^, ^26^ We hope to evaluate the sensitivities to various MLC errors using EPID and film dosimetry in future studies.

## 5 CONCLUSION

This study evaluated the MLC error sensitivities of various QA devices. Our results indicate that DD could be more useful than gamma analysis for daily MLC QA, and that a large‐area ionization chamber has a greater advantage for detecting systematic MLC error because of the large sensitive volume, while the other devices could not detect this error for some cases with a small range of MLC error.

## CONFLICTS OF INTEREST

The authors declare no conflict of interest.
